# Physical Exercise Performance in Temperate and Warm Environments Is Decreased by an Impaired Arterial Baroreflex

**DOI:** 10.1371/journal.pone.0072005

**Published:** 2013-08-07

**Authors:** Washington Pires, Samuel P. Wanner, Milene R. M. Lima, Ivana A. T. Fonseca, Ubirajara Fumega, Andrea S. Haibara, Cândido C. Coimbra, Nilo R. V. Lima

**Affiliations:** 1 Exercise Physiology Laboratory, Department of Physical Education, School of Physical Education, Physiotherapy and Occupational Therapy, Universidade Federal de Minas Gerais, Belo Horizonte, Minas Gerais, Brazil; 2 Department of Physiology and Biophysics, Institute of Biological Sciences, Universidade Federal de Minas Gerais, Belo Horizonte, Minas Gerais, Brazil; St. Joseph's Hospital and Medical Center, United States of America

## Abstract

The present study aimed to investigate whether running performance in different environments is dependent on intact arterial baroreceptor reflexes. We also assessed the exercise-induced cardiovascular and thermoregulatory responses in animals lacking arterial baroafferent signals. To accomplish these goals, male Wistar rats were subjected to sinoaortic denervation (SAD) or sham surgery (SHAM) and had a catheter implanted into the ascending aorta to record arterial pressure and a telemetry sensor implanted in the abdominal cavity to record core temperature. After recovering from these surgeries, the animals were subjected to constant- or incremental-speed exercises performed until the voluntary interruption of effort under temperate (25° C) and warm (35° C) conditions. During the constant-speed exercises, the running time until the rats were fatigued was shorter in SAD rats in both environments. Although the core temperature was not significantly different between the groups, tail skin temperature was higher in SAD rats under temperate conditions. The denervated rats also displayed exaggerated increases in blood pressure and double product compared with the SHAM rats; in particular, in the warm environment, these exaggerated cardiovascular responses in the SAD rats persisted until they were fatigued. These SAD-mediated changes occurred in parallel with increased variability in the very low and low components of the systolic arterial pressure power spectrum. The running performance was also affected by SAD during the incremental-speed exercises, with the maximal speed attained being decreased by approximately 20% in both environments. Furthermore, at the maximal power output tolerated during the incremental exercises, the mean arterial pressure, heart rate and double product were exaggerated in the SAD relative to SHAM rats. In conclusion, the chronic absence of the arterial baroafferents accelerates exercise fatigue in temperate and warm environments. Our findings also suggest that an augmented cardiovascular strain accounted for the early interruption of exercise in the SAD rats.

## Introduction

The physical exercise-induced increase in the demand of contracting muscles for oxygen and energetic substrates is a major challenge to body homeostasis and encompasses coordinated responses from the cardiovascular, ventilatory, hormonal, and thermoregulatory systems. To match the higher metabolic demands, landmark physiological responses, such as increases in heart rate (HR), mean arterial pressure (MAP), and the resetting of baroreflexes (which allows simultaneous increases in the HR and MAP), are usually observed immediately after exercise initiation [1,2,3]. The activation of the cardiovascular system occurs in parallel with the activation of the motor centers by a brain-mediated feed-forward mechanism (termed central command), which is integrated with the afferent stimuli from the muscle chemoreceptors (termed exercise pressor reflex) and from the arterial and cardiopulmonary baroreceptors. This integrated cardiovascular control provides physiological responses that match the requirements associated with a given exercise intensity [4].

The carotid and aortic baroreceptors buffer short-term fluctuations of blood pressure by modulating the brain stem-mediated autonomic outflow to the heart and blood vessels. These baroreceptors are involved in the cardiac and hemodynamic responses to exercise [5]. Previous reports showed that the surgical removal of the arterial baroafferents of the rat (sinoaortic denervation procedure - SAD) produced exaggerated exercise-induced increases in blood pressure [6,7] and iliac vascular conductance [8], and an exaggerated reduction in mesenteric conductance [9]. These investigations regarding the consequences of an impaired baroreflex sensitivity on the physiological responses to exercise are clinically relevant considering that the loss of sensitivity is an outcome common to many diseases, such as diabetes, obesity, metabolic syndrome, and hypertension [10,11,12], and also considering that physical exercise is a nonpharmacological tool for the treatment of such diseases [13,14].

Although there is substantial evidence demonstrating the role of the arterial baroreceptors in generating adequate autonomic-cardiovascular responses to exercise, no study has systematically investigated the effects of cardiovascular alterations induced by arterial barodenervation on prolonged physical performance. A theoretical model that has been recently used to explain exercise fatigue suggests that the interaction between an anticipatory feed-forward control and the afferent signals provided by peripheral receptors generates a conscious perception of effort, which regulates skeletal muscle recruitment and, consequently, exercise intensity [15,16,17]. However, the participation of afferent pathways in modulating physical performance is not universally accepted [18].

In response to exercises performed in a warm environment, the rates of heat dissipation must be greatly increased to avoid the occurrence of exertional hyperthermia, which may threaten survival. Therefore, aside from the high amounts of oxygenated blood and nutrients that are required in the working skeletal muscles, a higher percentage of the cardiac output is directed to the cutaneous vessels to dissipate the body heat [19]. It has been suggested that the higher and simultaneous requirement for blood in the muscles and cutaneous vessels increases the cardiovascular strain (one of the factors that modulates the rate of perceived exertion), which is the main explanation for the accelerated fatigue that occurs during prolonged exercise in warm conditions compared with temperate conditions [19]. Therefore, it is likely that the influence of surgically removing the arterial baroreflexes on exercise performance would be more evident under conditions of ambient thermal stress, a hypothesis that has not been investigated in previous experiments. Supporting this hypothesis, SAD exaggerated the increases in MAP, HR, mesenteric vascular resistance, and plasma norepinephrine concentrations when rats were exposed to a warm environment [20]. Therefore, the present study aimed to investigate whether prolonged exercise performance in temperate and warm environments is dependent on intact arterial baroreceptors and whether the loss of moment-to-moment regulation of blood pressure is associated with changes in cardiovascular and thermal regulation during physical exercise.

## Experimental Procedure

### Animals

Adult male Wistar rats weighing 280-350 g were used in all experiments. The animals were housed in individual cages under controlled light (lights on from 0500 until 1900 hours) and temperature (24 ± 1°C) conditions, with water and rat chow provided *ad libitum*. All experimental procedures were approved by the Ethics Committee of the Universidade Federal de Minas Gerais for the Care and Use of Laboratory Animals (protocol 178/10) and were carried out in accordance with the policies described in the Committee’s Guiding Principles Manual.

## Experimental Design

Three sets of experiments were conducted to achieve the goals of the present study. The first set was performed to investigate the impact of SAD on the cardiovascular and thermoregulatory responses during passive heating, an experimental approach in which hyperthermia is a consequence of the passive heat gained from the environment. The rats were subjected to a SAD or sham-denervation surgery (SHAM), and after recovering from these procedures for approximately three weeks, they were familiarized with the experimental setup and then underwent implantation of a temperature sensor in the abdominal cavity and an arterial catheter into the ascending aorta. Each animal was subjected to two experimental trials: exposure to temperate (25° C) and warm (35° C) environments.

The SAD-induced effects on the running performance and cardiovascular and thermoregulatory adjustments during constant-speed exercises (18 m/min, 5% inclination) were evaluated in the second set of experiments. Constant-speed exercises were conducted with the objective of promoting the same power output in both experimental groups. The rats were subjected to a SAD or SHAM surgery, and after recovering from these procedures, they were familiarized with running on a treadmill (five-day protocol) and then underwent implantation of a temperature sensor and an arterial catheter. Each animal was subjected to two exercise trials in the temperate and warm environments.

The third set was performed to determine the SAD effects on the maximal treadmill speed achieved during incremental exercises and to investigate whether the arterial baroreceptors influence cardiovascular responses at the maximal power output tolerated. The rats were initially familiarized with exercising on the treadmill and then subjected to an incremental-speed exercise to evaluate their innate running capacity and divide them into groups. On the following day, the animals were subjected to either a SAD or SHAM surgery. After recovering from these procedures, a second incremental exercise was performed. These two initial incremental exercises were performed at 25° C. Next, the animals underwent implantation of an arterial catheter and were again subjected to incremental exercises under the temperate and warm conditions.

This study was divided into three sets of experiments because the quality of the arterial pressure recording worsened after a few days. Each rat had two days to recover from the implantation of the arterial catheter [1] before being subjected to two experimental trials; a two-day interval was allowed between the trials. All experiments were performed between 0800 and 1600 hours, and care was taken to test the same animal at the same time of day. The experiments in the temperate environment were always performed prior to the experiments in the heat. A non-randomized experimental design was selected because of a concern regarding the possible occurrence of heat-related disorders after running at 35° C, which would have prevented us from subjecting the rats to the subsequent exercise in the temperate ambient and measuring their blood pressures. There is evidence that rats cannot restore their normal core body temperature (T_core_) circadian rhythm during the 10 days that follow a severe heat exposure [21].

### Treatment of the Animals

#### Anesthesia and postoperative care

All surgical procedures were performed under ketamine-xylazine anesthesia (90 and 10.5 mg/kg body mass, respectively, i.p.). Moreover, immediately after the surgeries, the rats received an intramuscular prophylactic dose of antibiotics (pentabiotic, 48,000 IU/kg) and a subcutaneous injection of analgesic medication (flunixin meglumine, 1.1 mg/kg).

#### Sinoaortic denervation

The sinoaortic denervation was performed according to the method described by Krieger [22]. Briefly, a midline incision was made in the ventral region of the neck, and the bilateral sternocleidomastoideus and omohyoideus muscles were reflected laterally to expose the common carotid arteries. The aortic nerves were identified with the help of a surgical binocular microscope (Opto FI04, São Carlos, SP, Brazil) and isolated, and the nerves were then bilaterally sectioned. The sympathetic trunks were bilaterally sectioned below the superior cervical sympathetic ganglion, and the superior laryngeal nerves were bilaterally sectioned as close to the larynx as possible to interrupt any other aortic nerve filaments. The fibers and connectives tissues of the wall of the carotid artery, including the carotid bodies, were stripped around the bifurcation area on both sides, and the area was painted with a small amount of phenol (10% diluted in ethanol). As a control procedure, some rats were subjected to a sham surgery. An incision was made in the neck, and all the nerves described above were exposed without being sectioned.

At the end of all experimental trials, a venous catheter was implanted into the right jugular vein for the administration of vasoactive drugs with the objective of testing the effectiveness of barodenervation. Baroreflex sensitivity was assessed by examining the cardiac reflex response to increases and decreases in MAP induced by bolus intravenous injections of phenylephrine (1.0-2.5 µg/mL in 0.1 mL of saline) and sodium nitroprusside (2-5 µg/mL in 0.1 mL of saline), respectively. Sinoaortic denervation reduced baroreflex sensitivity to both phenylephrine and sodium nitroprusside by approximately 95% (Table 1). The SAD rats also presented a higher HR during rest and a more pronounced decrease in MAP in response to sodium nitroprusside compared with the SHAM group. Taken together, these responses indicate that our sinoaortic denervations were effective [23].

**Table 1:** Resting values of the cardiovascular parameters and the baroreflex sensitivity to intravenous injection of vasoactive agents in rats subjected to sinoaortic denervation (SAD) or sham surgery (SHAM).

Parameters	SHAM (*n* = 14)	SAD (*n* = 14)
Mean arterial pressure (mmHg)	113 ± 3	112 ± 5
Heart rate (bpm)	358 ± 12	426 ± 13**
*Intravenous phenylephrine*		
Δ mean arterial pressure (mmHg)	36 ± 3	41 ± 4
Reflex bradycardia (bpm)	-92 ± 12	-5 ± 2**
Baroreflex sensitivity (bpm^-1^∙mmHg)	-2.51 ± 0.14	-0.12 ± 0.06**
*Intravenous sodium nitroprusside*		
Δ mean arterial pressure (mmHg)	-26 ± 2	-46 ± 6*
Reflex tachycardia (bpm)	68 ± 7	3 ± 2**
Baroreflex sensitivity (bpm^-1^∙mmHg)	-2.77 ± 0.32	-0.10 ± 0.06**

Values are means ± SEM. *P < 0.01 and **P < 0.001 compared with the SHAM group.

### Familiarization with the treadmill running exercise

The rats were gradually encouraged to exercise on a treadmill designed for small animals (Modular Treadmill, Columbus Instruments, OH, USA) by light electrical stimulation (0.5 mA). After resting for 5 min on the treadmill belt, the rats were made to run at a constant speed of 18 m/min at a 5% inclination for 5 min. This familiarization protocol was conducted across five consecutive days [24]. The purpose of these preliminary exercise sessions was to show the animals which direction to run, without becoming entangled in the skin thermocouple wires. The rats that were unable to run well during the familiarization sessions (i.e., the rats frequently exposed to the electrical stimulation at the end of the treadmill belt) were excluded from the study.

### Surgical implantations of an arterial catheter and a temperature sensor

Following the last familiarization exercise session, a catheter was surgically implanted in the rats for measurement of the pulsatile arterial pressure. A polyethylene catheter (PE-10 connected to a PE-50; Becton Dickinson, Franklin Lakes, NJ, USA), filled with heparin diluted in isotonic saline, was inserted into the left common carotid artery. The free end of the PE-50 tubing was tunneled subcutaneously and exteriorized at the cervical dorsal area [1]. Immediately after the arterial cannulation, a temperature sensor (TR3000 XM-FM; Vital View Mini-Mitter, Sunriver, OR, USA) was implanted in the peritoneal cavity. A small incision was made in the linea alba of the abdominal muscle, the peritoneal cavity was exposed, and the telemetry transmitter was inserted. After the insertion, the abdominal muscle and skin were sutured in layers.

### Passive heating

On the day of the experiments, each rat was weighed, a thermocouple (YSI Inc., Dayton, OH, USA) was fixed to its tail surface, the arterial cannula was connected to a pressure transducer (Biopac Systems, Santa Barbara, CA, USA), and the rat was placed inside an acrylic chamber (49.5 cm long x 14 cm wide x 13.5 cm high). The pressure transducer was coupled to an A/D Data Acquisition System (MP100, Biopac Systems). An electrical fan positioned at one end of the chamber generated an airflow rate of 2.0-2.5 m/min. The animals were allowed to move freely in their home cages for 60 min in a temperate environment (25° C). After T_core_ and tail skin temperature (T_skin_) values had stabilized, the rats were kept in the temperate environment for an additional 60 min or were passively heated. To heat the environment inside the chamber (35° C), an electrical heater (Britânia model AB 1100; Curitiba, PR, Brazil) was positioned at the same level, 20-30 cm from the fan, and turned on at 1200 W [25]. The thermoregulatory and cardiovascular responses were measured throughout these experiments.

### Constant-speed exercises

The ambient temperature (T_a_) inside the treadmill chamber was set at 25 or 35°C. The thermocouple was fixed to the rat’s tail with tape, and the arterial catheter was connected to the pressure transducer. Then, the animals were subjected to treadmill running at a constant speed of 18 m/min and an inclination of 5%. The exercise was performed until the animals were fatigued, which was defined as the point at which the animals were no longer able to keep pace with the treadmill for at least 10 s, even when being stimulated by the light electrical stimuli [24].

### Incremental-speed exercises

The experimental procedures were similar to those described in the previous section. However, the thermoregulatory parameters were not measured and, instead of running at a constant speed of 18 m/min, the rats were subjected to incremental speed-exercises. During the first 2 min, the rats ran at 10 m/min, followed by increments of 1 m/min every 2 min until they were fatigued [26].

### Measures and calculations

The intraperitoneal temperature was established as the T_core_ index and was measured by telemetry. T_skin_ was measured using a thermocouple attached to the lateral surface ^≈^1 cm from the base of the tail. To measure T_a_, a thermocouple was fixed to the ceiling of the treadmill chamber. T_core_ values were recorded every 10 s, whereas T_skin_ and T_a_ inside the treadmill were measured every minute during the experimental trials. The HR, MAP, systolic arterial pressure (SAP), and diastolic arterial pressure (DAP) values were obtained from pulsatile arterial pressure recordings with the AcqKnowledge 3.7.0 software (Biopac Systems). The double product, an index of the myocardial work, was calculated by multiplying SAP by HR. To analyze the lability of T_skin_ and MAP, we calculated their average deviation values throughout the resting experiments under temperate conditions.

### Systolic arterial pressure and heart rate variability analysis

The tape-recorded arterial pressure signal was sampled at 2 kHz. The SAP values were identified beat by beat, and the pulse interval was computed as the interval between two consecutive systolic peaks using a customized routine (MATLAB 7.8, Mathworks, Natick, MA, USA). Time- and frequency-domain analyses were evaluated during the passive heating protocol using a 30-min period selected from continuous recording after the stabilization of the cardiovascular parameters. In the exercising rats, we analyzed the recording during the 6-min period that preceded the interruption of the effort. This shorter period was selected because the SAD rats presented a short running time to fatigue in the heat (only 14 ± 1 min). The power spectral density was obtained by fast Fourier transformation and Hanning windows (512) with 50% overlap. The spectral power components for very low- (VLF, from 0.0195 to 0.25 Hz), low- (LF, from 0.27 to 0.74 Hz), and high-frequency (HF, from 0.76 to 5 Hz) bands were evaluated. These bandwidths were previously used to analyze the spectrum of the blood pressure and HR variability in chronic SAD rats [27]. The power spectrum density integration within each frequency bandwidth was obtained with the aid of software (Cardioseries v.2.2, São Paulo, SP, Brazil).

### Statistical analysis

The data are expressed as the means ± SEM. The baroreflex sensitivity was compared between experimental groups (SAD vs. SHAM) using unpaired Student’s *t*-tests. The parameters associated with the blood pressure and HR variability and the physical performance indexes (maximal treadmill speed and total exercise time) were compared between groups and Ta by two-way analysis of variance (ANOVA), with repeated measures applied only for the factor Ta. The cardiovascular and thermoregulatory responses that were continuously measured throughout the experiments were compared between groups, Ta and across time points by three-way ANOVA. The *post hoc* Student-Newman-Keuls test was used for multiple comparisons. The curves that describe the percentage of running rats along exercise time points were compared using the logrank test [28]. The significance level was set at *P* < 0.05.

## Results

### Effects of SAD on the cardiovascular and thermoregulatory adjustments in resting rats under temperate and warm conditions

**Figure 1:**
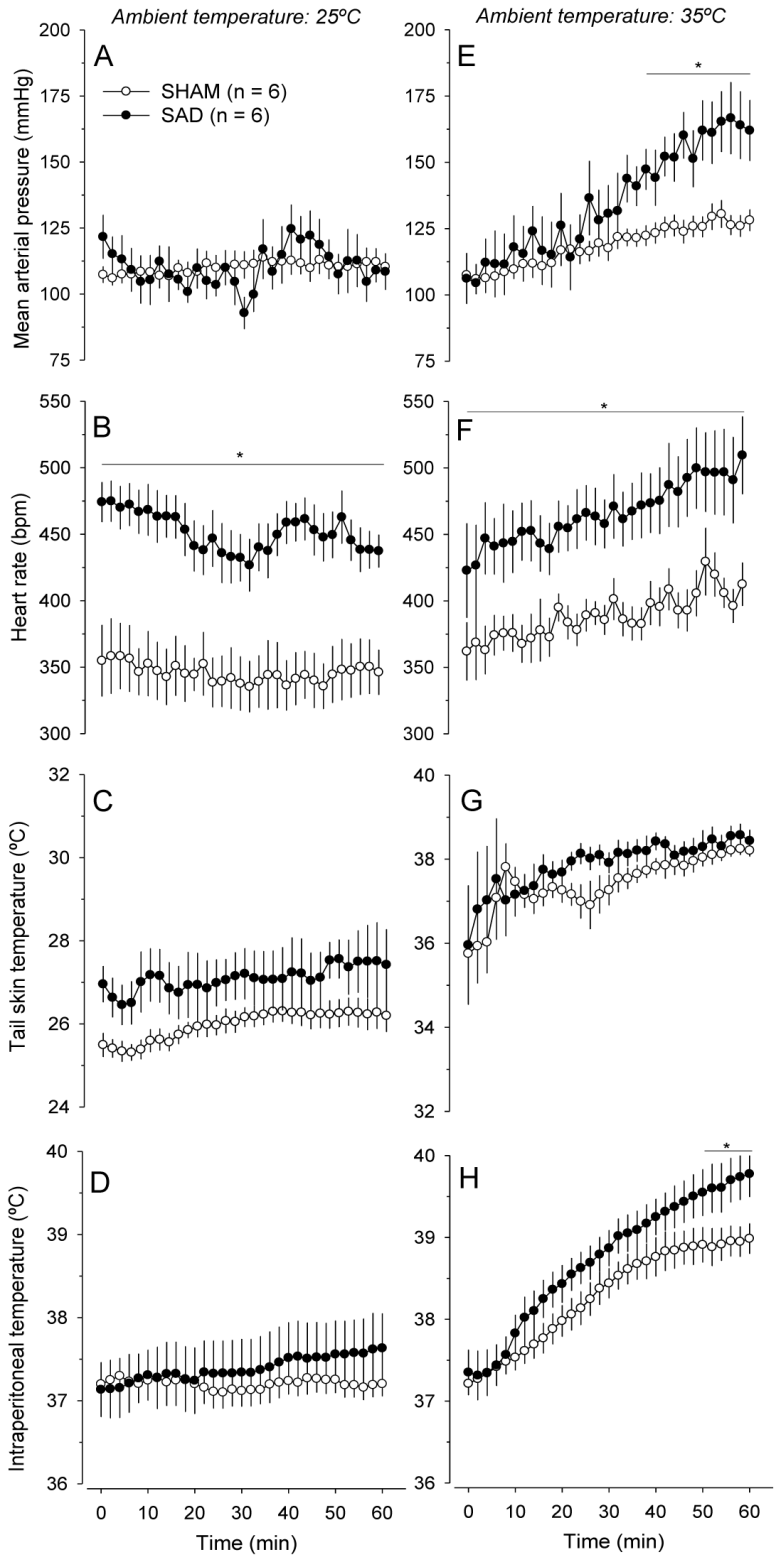
Cardiovascular and thermoregulatory responses induced by passive heating. Effects of the sinoaortic denervation (SAD; n = 6) or sham surgery (SHAM; n = 6) on the mean arterial pressure (*A* and *E*), HR (*B* and *F*), tail skin temperature (*C* and *G*), and intraperitoneal temperature (*D* and *H*) of rats that were allowed to rest in a temperate environment (25° C) or were exposed to heat (35° C). The values are means ± SEM. * *P* < 0.05 compared with the sham group.

Under resting conditions in the temperate environment, the mean values of the MAP were not altered by denervation (Figure 1A). However, the blood pressure lability determined by calculating the standard deviation of the MAP during the 60 min of rest was three-fold higher in the SAD compared with SHAM animals (15 ± 3 mmHg vs. 5 ± 1 mmHg; *P* < 0.05). In addition, the HR was higher in the SAD compared with SHAM animals throughout the resting period (437 ± 12 bpm vs. 346 ± 17 bpm at the 60^th^ min; *P* < 0.05; Figure 1B). The mean values of T_skin_ and T_core_ were not different between the experimental groups (Figure 1C and D), although the T_skin_ lability was higher in the SAD compared with SHAM rats (0.73 ± 0.09°C vs. 0.40 ± 0.06°C; *P* < 0.05; Figure 1C).

The 60 min of heat exposure increased the MAP, T_skin_ and T_core_ in both experimental groups (*P* < 0.001 for the three parameters). SAD markedly enhanced the cardiovascular responses induced by passive heating, including an exaggerated the increase in MAP compared with the SHAM condition, as illustrated in Figure 1E (162 ± 11 mmHg vs. 128 ± 4 mmHg at the end of the passive heating; *P* < 0.05). As observed in the temperate environment, the HR was higher in the SAD than in the SHAM rats throughout the passive heating protocol (Figure 1F). Our data are in accordance with the results of an earlier study conducted by Kregel et al. [20], who demonstrated three- to four-fold greater passive heating-induced increases in the MAP and HR in SAD rats.

The cutaneous heat loss through the tail vessels was not different between the experimental groups (Figure 1G); however, at the end of passive heating, T_core_ was ~0.8°C higher in the SAD animals compared with the controls (39.8 ± 0.3°C vs. 39.0 ± 0.2°C; *P* < 0.05; Figure 1H). A novel finding of the present study is that the inability to protect T_core_ against heat caused by SAD is not the consequence of impaired cutaneous heat loss. Moreover, our findings also suggest that the exaggerated sympathetic outflow of denervated rats is most likely site-specific because it was not increased in the skin vessels, as evidenced by similar T_skin_ responses in the animals from both groups (Figure 1G).

Table 2 presents the parameters associated with the variabilities of the SAP and HR during exposure to temperate and warm environments. As can be inferred from the standard deviation values, the SAD significantly increased the SAP variability at both T_a_, without affecting the HR variability. In the temperate environment, the power spectral density for the VLF component of the SAP variability spectrum was higher, whereas the VLF and LF components of the HR variability were lower, in the SAD compared with the control animals. These observations corroborate the findings from previous investigations [27,29], including the study of Dworkin [30], which demonstrated that the maximum baroreflex effectiveness occurs in the VLF range. During passive heating, all three components of the SAP variability were significantly increased by SAD. In contrast, the VLF and HF components of the HR variability were lower in the SAD compared with control rats. These responses are in concordance with recent findings showing that the HR variability is primary generated by baroreflex-mediated modulation of cardiac autonomic outflows [31].

**Table 2:** Power spectrum density of the systolic arterial pressure and HR variability in rats subjected to the SAD or SHAM surgery.

	Temperate (25°C)		Warm (35°C)	
Parameters in freely moving rats	SHAM (n = 6)	SAD (n = 6)	SHAM (n = 6)	SAD (n = 6)
*Systolic arterial pressure variability*				
Systolic pressure (mmHg)	128 ± 6	127 ± 6	141 ± 4	160 ± 10+*
S.D. (mmHg)	4.8 ± 0.4	19.1 ± 3.8*	7.4 ± 1.1+	24.7 ± 2.0*
VLF component (mmHg^2^)	7.8 ± 0.6	34.1 ± 8.6*	10.6 ± 2.3	46.7 ± 6.3*
LF component (mmHg^2^)	1.9 ± 0.6	3.2 ± 0.7	5.8 ± 1.1+	10.6 ± 1.6+*
HF component (mmHg^2^)	1.3 ± 0.2	1.9 ± 0.1	2.6 ± 0.8	12.6 ± 6.7+*
*Heart rate variability*				
Pulse interval (ms)	175 ± 11	134 ± 5*	151 ± 5	122 ± 6*
S.D. (ms)	6.5 ± 1.0	5.9 ± 0.6	10.1 ± 1.3+	7.4 ± 1.0
VLF component (ms^2^)	8.4 ± 2.0	1.2 ± 0.3*	5.4 ± 1.4	2.2 ± 0.4*
LF component (ms^2^)	1.4 ± 0.3	0.5 ± 0.2*	1.3 ± 0.3	0.7 ± 0.1
HF component (ms^2^)	8.0 ± 1.4	5.1 ± 0.6	11.7 ± 1.7	9.3 ± 0.6

These parameters were calculated in rats resting in a temperate environment (25° C) or exposed to heat (35° C).

S.D. = standard deviation; VLF = very low frequency; LF = low frequency; HF = high frequency

Values are means ± SEM

^*^ *P* < 0.05 compared with the SHAM group (in the same environment).

+ P < 0.05 compared with the temperate environment (for the same experimental group).

### Effects of SAD on the running performance and on the cardiovascular and thermoregulatory adjustments during constant-speed exercises in temperate and warm environments

**Figure 2:**
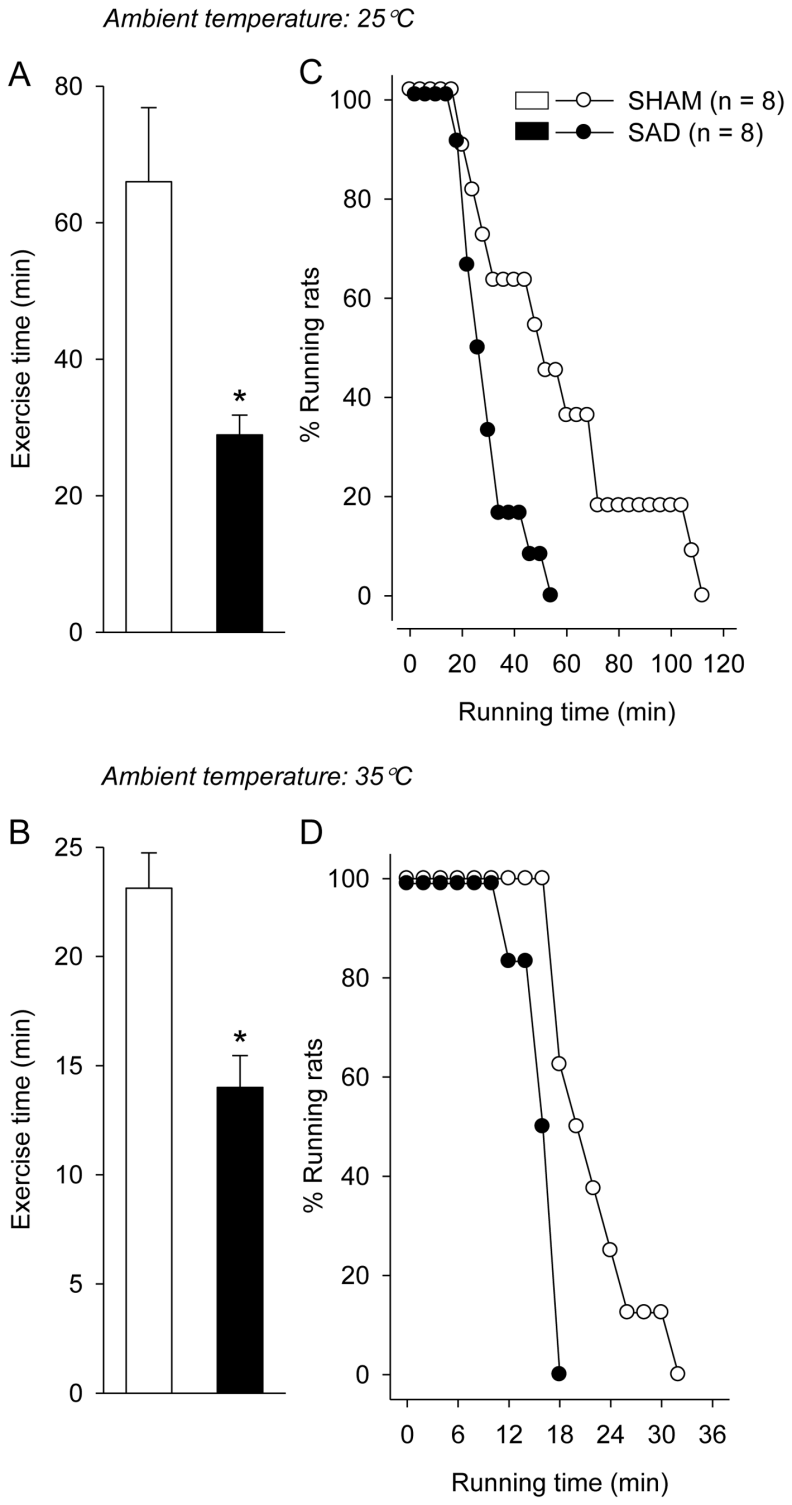
Running performance during constant-speed exercises in temperate and warm environments. Effects of the sinoaortic denervation (SAD; n = 8) or sham surgery (SHAM; n = 8) on running time until the voluntary interruption of the effort (A and B) during constant-velocity exercises (18 m/min). The exercises were performed in temperate (25° C) and warm (35° C) environments. The values represent the means ± SEM. * *P* < 0.01 compared with the SHAM group. Panels C and D show the curves of the maximal exercise duration tolerated by the SAD or SHAM rats subjected to constant-speed exercises at the two ambient temperatures. The data are expressed as proportions of rats that were still running at given time points.

As shown in Figure 2A, SAD markedly reduced running time to fatigue by 56% during the constant-speed exercise in the temperate environment (29 ± 3 min for SAD rats vs. 66 ± 11 min for SHAM rats; *P* < 0.01). As expected, the running performance in the warmer environment was significantly decreased by 66 ± 9% and 52 ± 6% in the SHAM and SAD rats, respectively, compared with their performance in the temperate environment (Figure 2A and B *; P* < 0.05 for both groups). Therefore, the running performance of SAD rats was also lower compared with that of the control rats in the warm environment (14 ± 1 min vs. 23 ± 2 min; *P* < 0.001). The logrank analysis also revealed the ergolytic effects caused by the SAD in both environments (*P* < 0.001 for 25° C and 35° C; Figure 2C and D). When all SAD rats had interrupted their exercise in the temperate environment, 45% of the SHAM animals were still running. Moreover, when 62% of the SHAM rats were still running in the warm environment, all animals in the SAD group had already fatigued.

**Figure 3:**
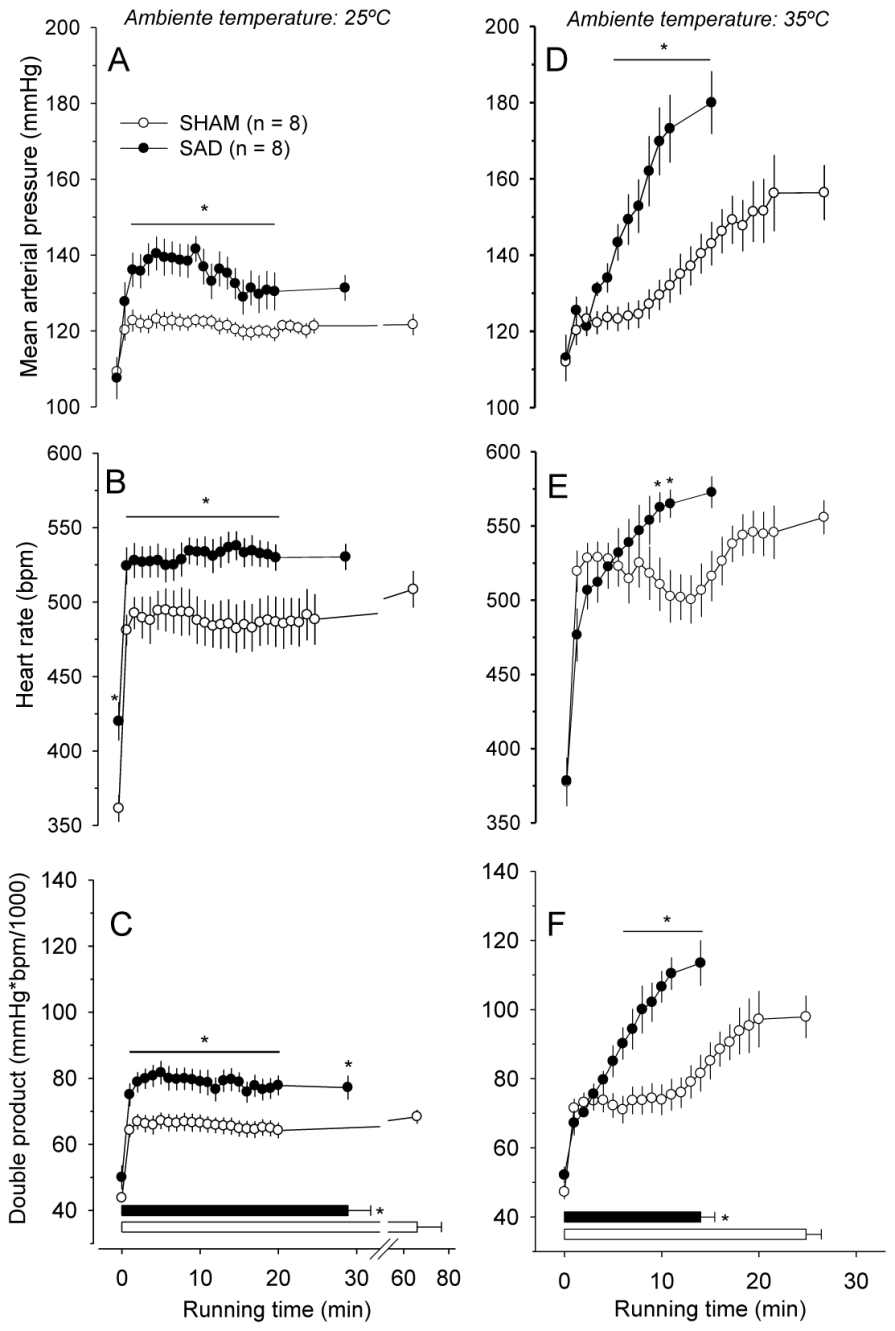
Cardiovascular responses induced by constant-speed exercises in temperate and warm environments. Temporal profile of the exercise-induced changes in the MAP (*A* and *D*), HR (*B* and *E*), and double product (*C* and *F*) in rats that were previously subjected to the sham surgery (*n* = 8) or sinoaortic denervation (*n* = 8). The constant-speed exercises (18 m/min) were performed in temperate (25° C) and warm (35° C) environments. The values represent the means ± SEM. The times until the interruption of the effort are indicated by the horizontal bars. * *P* < 0.05 compared with the SHAM group.

During the constant-speed exercise at 25° C, SAD enhanced the exercise-induced increases in MAP (142 ± 3 mmHg vs. 123 ± 2 mmHg; *P* < 0.05; Figure 3A), HR (534 ± 9 bpm vs. 488 ± 16 bpm; *P* < 0.05; Figure 3B), and double product (81± 4 mmHg.bpm/1000 vs. 66 ± 3 mmHg.bpm/1000; *P* < 0.05; Figure 3C) compared with the control group (all three datasets correspond to the 10^th^ min of exercise). Irrespective of the experimental group, constant-speed exercises in the warm environment produced significant increases in MAP, HR and double product compared with the temperate environment; these differences were particularly observed at the voluntary interruption of effort. Moreover, at 35° C, the cardiovascular parameters were also higher in the SAD compared with control rats (MAP: 173 ± 9 mmHg vs. 132 ± 5 mmHg, Figure 3D; HR: 565 ± 9 bpm vs. 503 ± 18 bpm, Figure 3E; comparisons made at the 10^th^ min of exercise; both *P* < 0.05). One remarkable observation was that the combination of passive heating with treadmill running greatly exaggerated the effects of SAD on the MAP; the differences between the groups persisted until the end of exercise, and although the SAD rats ran for only an average of 14 min, their MAP values were higher than 180 mmHg when the effort was interrupted.

**Figure 4:**
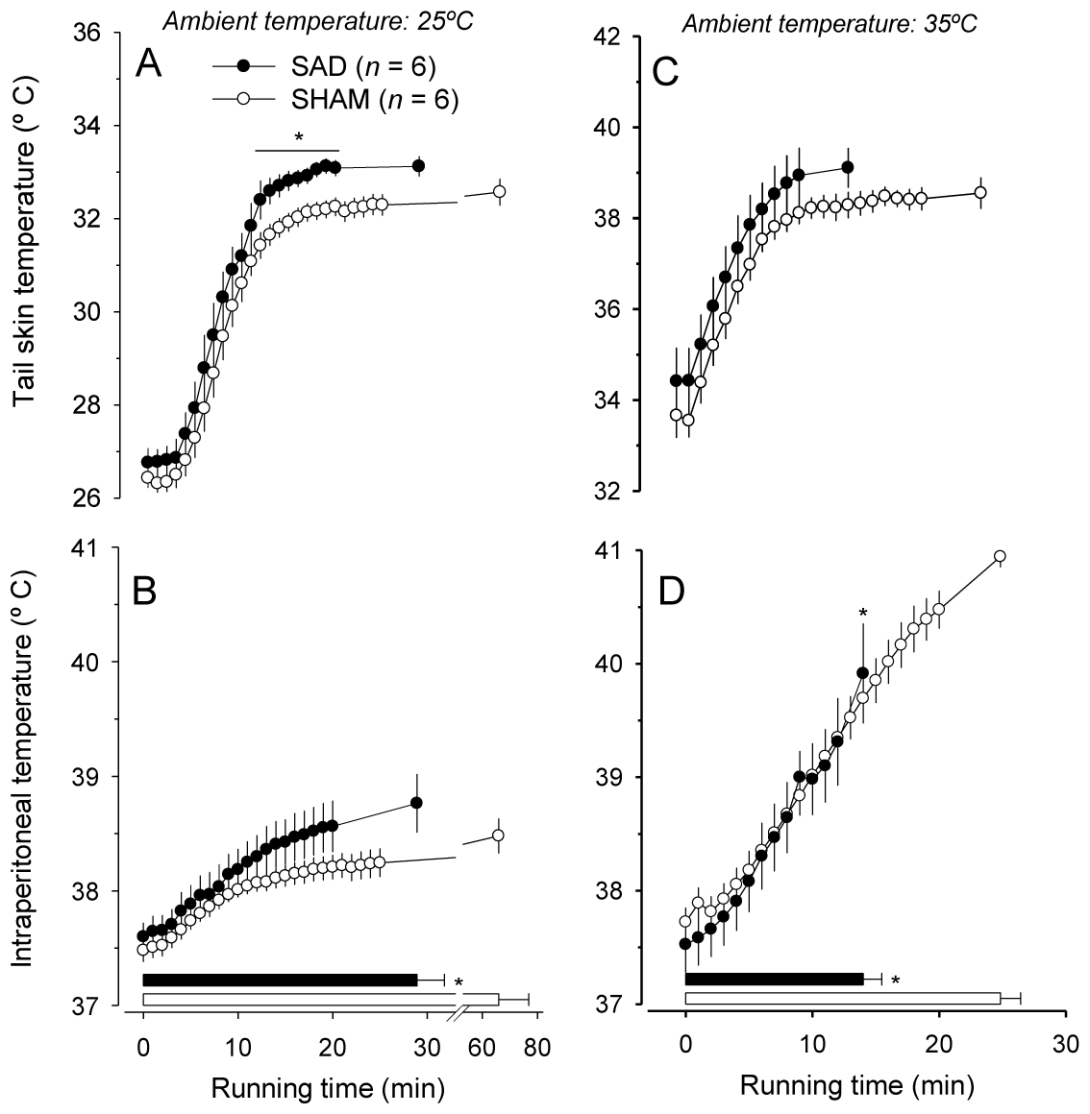
Thermoregulatory responses induced by constant-speed exercises in temperate and warm environments. Temporal profile of exercise-induced changes in the tail skin temperature (*A* and *C*) and intraperitoneal temperature (*B* and *D*) in rats that were previously subjected to the sham surgery (*n* = 8) or sinoaortic denervation (*n* = 8). The constant-speed exercises (18 m/min) were performed in temperate (25° C) and warm (35° C) environments. The values represent the means ± SEM. The times until the interruption of the effort are indicated by the horizontal bars. * *P* < 0.05 compared with the SHAM group.

Regarding the thermoregulatory responses at 25° C, T_skin_ was significantly higher in the SAD rats compared with the SHAM rats from the 12^th^ to the 20^th^ min of exercise (Figure 4A), while the T_core_ was not affected by the denervation procedure (Figure 4B). The fact that SAD rats exhibited higher T_skin_ and the same T_core_ suggests that these animals counteracted a higher running-induced heat production by increasing the cutaneous heat loss. The evidence for increased heat production in the SAD rats is further corroborated by their higher HR throughout the exercise (Figure 3B). In contrast, there were no significant differences in the exercise-induced increase in T_skin_ and T_core_ between the two groups at 35° C (Figure 4C and D); the only exception was the lower T_core_ in the SAD compared with control animals at the interruption of effort (39.9 ± 0.4°C vs. 40.9 ± 0.1°C; *P* < 0.05; Figure 4D).

As shown in Table 3, during the constant-speed exercise in temperate and warm environments, the VLF and LF components of the SAP variability were higher in the SAD compared with SHAM rats. In contrast, the HR variability was not significantly affected by SAD during the constant-speed treadmill running.

**Table 3:** Power spectrum density of the systolic arterial pressure and HR variability in rats subjected to the SAD or SHAM surgery.

	Temperate (25°C)		Warm (35°C)	
Parameters in running rats	SHAM (n = 8)	SAD (n = 8)	SHAM (n = 8)	SAD (n = 8)
*Systolic arterial pressure variability*				
Systolic pressure (mmHg)	142 ± 2	138 ± 6	178 ± 6+	190 ± 10+
S.D. (mmHg)	4.1 ± 0.2	8.2 ± 1.0*	8.1 ± 0.7+	14.8 ± 1.1+*
VLF component (mmHg^2^)	2.7 ± 0.4	20.3 ± 4.1*	5.9 ± 0.8+	26.9 ± 6.2*
LF component (mmHg^2^)	3.9 ± 0.6	7.3 ± 1.2*	5.9 ± 1.1	8.6 ± 1.5
HF component (mmHg^2^)	7.2 ± 1.2	11.8 ± 3.3	15.1 ± 3.3+	17.0 ± 4.3+
*Heart rate variability*				
Pulse interval (ms)	124 ± 3	110 ± 4*	103 ± 4+	107 ± 4
S.D. (ms)	4.8 ± 0.2	5.6 ± 0.3	5.6 ± 0.7	7.0 ± 1.2
VLF component (ms^2^)	0.7 ± 0.2	1.0 ± 0.3	0.6 ± 0.2	0.4 ± 0.2
LF component (ms^2^)	0.6 ± 0.2	0.8 ± 0.1	0.3 ± 0.1	0.5 ± 0.2
HF component (ms^2^)	16.3 ± 3.8	22.3 ± 2.8	12.6 ± 3.1	18.6 ± 4.0

The parameters were calculated in rats subjected to constant-velocity exercises in temperate (25° C) and warm (35° C) environments.

S.D. = standard deviation; VLF = very low frequency; LF = low frequency; HF = high frequency

Values are means ± SEM

^*^ *P* < 0.05 compared with the SHAM group (in the same environment).

+ P < 0.05 compared with the temperate environment (for the same experimental group).

### Effects of SAD on running performance and cardiovascular adjustments during incremental-speed exercises in temperate and warm environments

**Figure 5:**
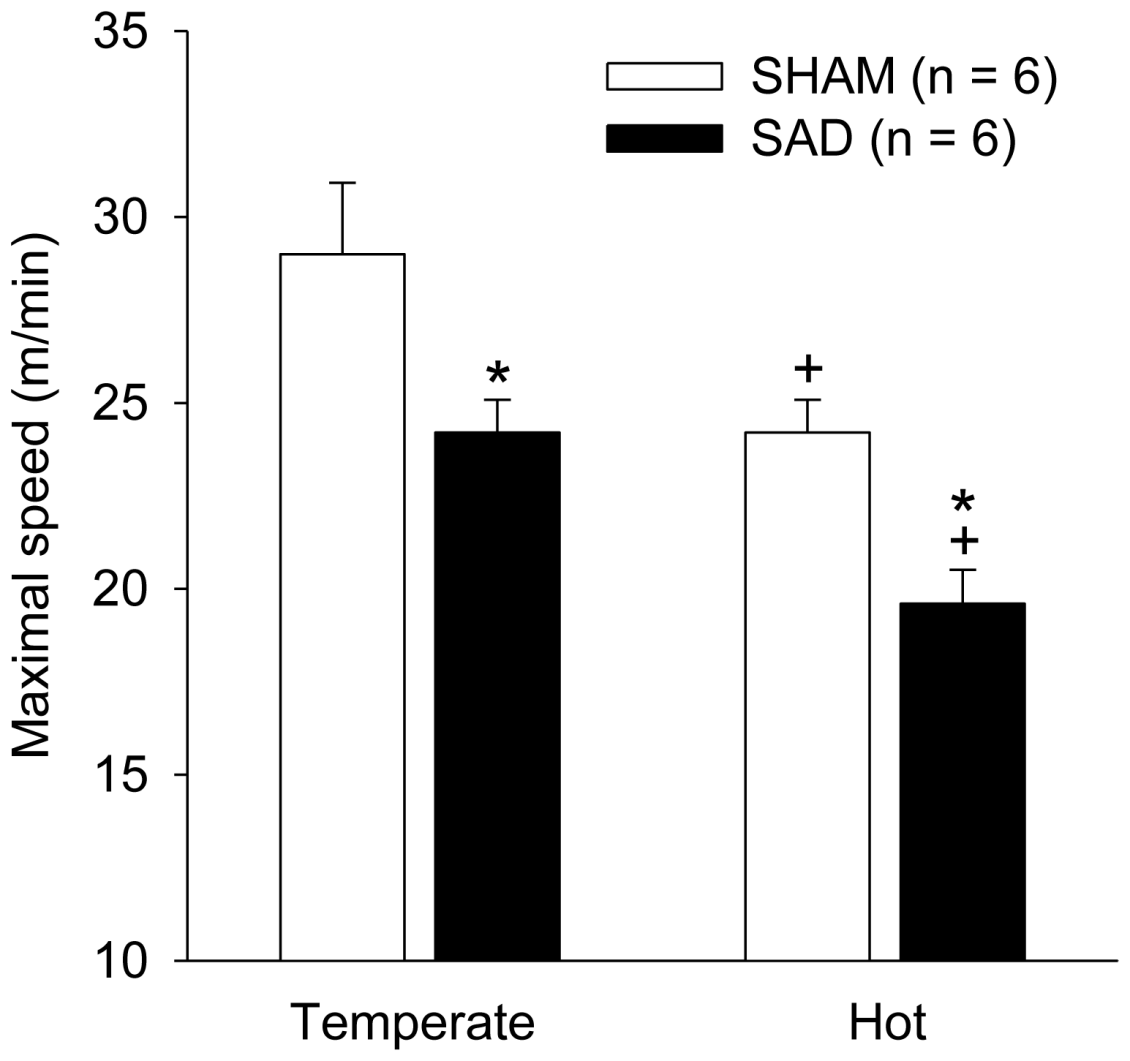
Running performance during incremental-speed exercises in temperate and warm environments. Effects of sinoaortic denervation (SAD; n = 6) or sham surgery (SHAM; n = 6) on the maximal speed achieved by rats during the incremental-velocity exercises. The exercises were performed in temperate (25° C) and warm (35° C) environments. The values are means ± SEM. + *P* < 0.05 compared with the temperate condition. * *P* < 0.05 compared with the SHAM group.

SAD significantly reduced the maximal speed achieved at 25° C by approximately 20% (25 ± 1 m/min after SAD vs. 31 ± 1 m/min before SAD; *P* < 0.05; Table 4). Interestingly, the running performance was not affected by the implantation of arterial and venous catheters. As expected, a heat-related impairment in performance was observed in both groups (Figure 5). In the warm environment, the SHAM and SAD rats showed, respectively, 21% and 17% reductions in performance compared with their running capacity in the temperate environment. Aside from these heat-mediated decreases in physical performance, the SAD rats attained lower maximal speeds than the SHAM rats during the incremental exercise at 35° C (20 ± 1 m/min vs. 23 ± 1 m/min; *P* < 0.05; Figure 5).

**Table 4:** Changes in the running performance induced by SAD or SHAM surgery during incremental-speed exercises in temperate (25° C) and warm (35° C) environments.

	Maximal treadmill speed (m/min)	
Experimental conditions	SHAM (n = 6)	SAD (n = 6)
Before SAD or SHAM surgery at 25°C	30 ± 1	31 ± 1
After SAD or SHAM surgery at 25°C	30 ± 1	25 ± 1*
After arterial and vein cannulations	29 ± 2	24 ± 1*

Values represent the means ± SEM. After the rats had recovered from the SAD or SHAM surgery, the body masses of the SAD and SHAM rats were 311 ± 14 and 329 ± 13 g, respectively. *P<0.05 compared with the SHAM group.

**Figure 6:**
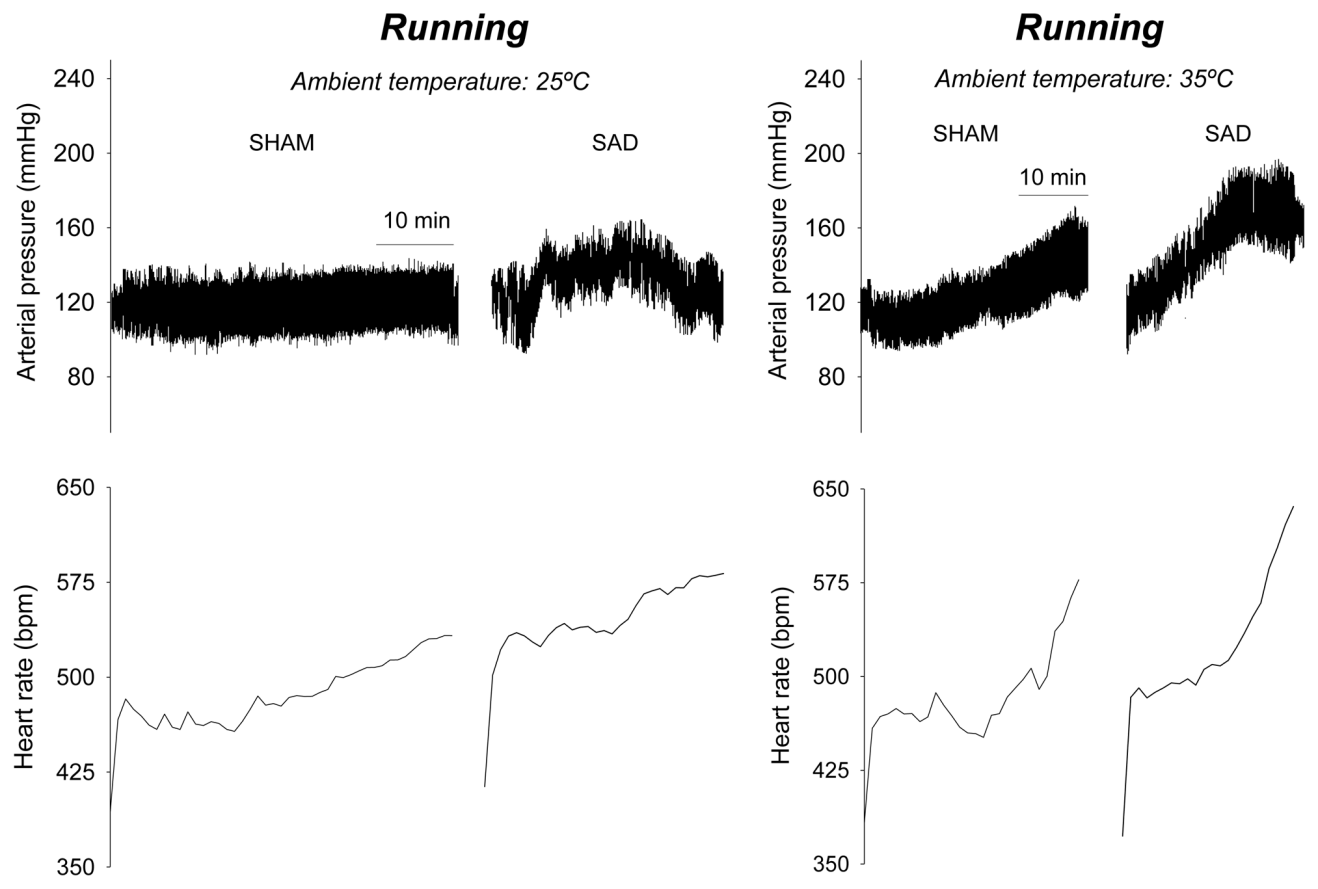
Cardiovascular recordings during incremental-speed exercises in temperate and warm environments. Representative recordings of the MAP and HR of rats during the incremental-speed exercises in temperate (25° C) and warm (35° C) environments. The recordings were obtained from rats that were previously subjected to the SHAM or SAD surgery.

**Figure 7:**
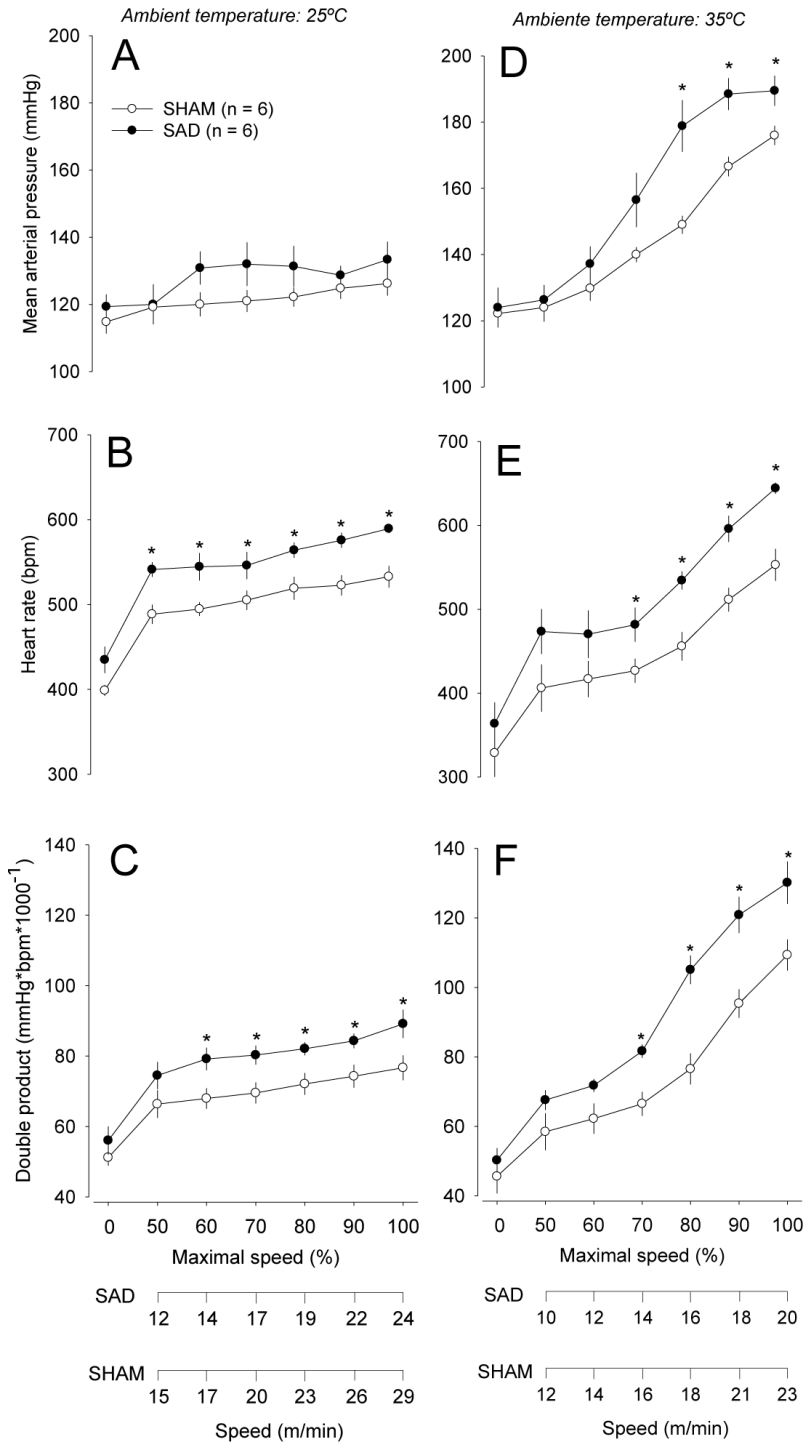
Cardiovascular responses induced by incremental-speed exercises in temperate and warm environments. Changes in the systolic arterial pressure (*A* and *D*), HR (*B* and *E*), and double product (*C* and *F*) induced by incremental-speed exercise in rats that were previously subjected to the sham surgery (*n* = 6) or chronic sinoaortic denervation (*n* = 6). The cardiovascular parameters were plotted against the percentage of maximal speed attained. The data were individually normalized to each animal’s maximal speed. The absolute treadmill speeds corresponding to the percentage of maximal speeds attained by the animals from both groups are represented at the bottom of the figure. The values are means ± SEM. * *P* < 0.05 compared with the SHAM group.

Figure 6 depicts the pulsatile arterial pressure and HR recordings in a representative rat from each experimental group. The recordings suggest that the incremental running induced higher cardiovascular strain in the SAD rats, as evidenced by their maximal HR achieved at 25 and 35°C. These individual values were used to perform the analysis presented in Figure 7, in which the cardiovascular responses in the SAD and SHAM rats are plotted against the percentage of the maximal power output tolerated by each individual animal.

At 25° C, the increase in the HR was more pronounced in the SAD animals from 50% of the maximal speed achieved during the incremental exercise to the maximal speed (maximal HR: 593 ± 5 bpm for SAD rats vs. 533 ± 13 bpm for SHAM rats; *P* < 0.05; Figure 7B). Thus, the cardiac workload (as inferred from the double product values) was increased in the SAD compared with SHAM rats from 60% of the maximal speed to the maximal speed (89 ± 4 bpm·mmHg/1000 vs. 77 ± 3 bpm·mmHg/1000 at the maximal speed; *P* < 0.05; Figure 7C). As previously observed for constant-speed exercises, the running-induced increases in MAP and double product were also exaggerated by the warm ambient during the incremental exercises. In addition, the magnitude of the MAP and HR responses in the SAD animals was higher than that in the SHAM animals (Figure 7D and E), also leading to higher double product values in denervated rats running at 35° C (130 ± 6 bpm·mmHg/1000 vs. 109 ± 4 bpm·mmHg/1000 at the maximal speed; *P* < 0.05; Figure 7F).

## Discussion

In summary, our results demonstrated that SAD rats subjected to treadmill running presented: 1) accelerated fatigue during constant- and incremental-speed exercises in both temperate and warm environments; 2) exaggerated cardiovascular responses (MAP, HR and double product) for given absolute or relative exercise intensities and at the maximal power output tolerated; 3) increased cutaneous heat loss during the constant-speed exercise at 25° C; and 4) increased SAP variability without changes in the HR variability.

A novel finding of this study was that SAD shortened the running time until the voluntary interruption of effort by 20 to 56% in all four experimental conditions studied (Figures 2 and 5). Exercise fatigue has been considered a brain-controlled, multi-factorial sensation that acts as a protective mechanism that regulates exercise intensity or even the ability to keep exercising [15,32]. During the constant-speed exercises, the lower performance of the SAD rats was accompanied by higher exercise-induced increases in the MAP, HR, and double product (Figure 3). However, considering the fact that the SAD rats were actually exercising at higher relative intensities than SHAM rats, we cannot correlate, without caveats, the early interruption of the effort with any exaggerated cardiovascular response; the SAD rats most likely ran less because they exerted a more intense effort. This methodological issue was overcome by conducting the incremental-speed exercises. At the maximal power output tolerated, the SAD rats presented higher HR and double product values, although the SAD decreased the maximal power output by 20%. It is widely acknowledged that cardiopulmonary factors, such as HR, cardiac output and respiration, are linked to conscious sense of effort [16,32]. Thus, our findings are suggestive that the exaggerated cardiovascular responses can to explain the ergolytic effects in SAD animals. In addition, considering the exercise fatigue as a protection mechanism, the greater cardiovascular parameters in the SAD rats at fatigue, provide an evidence of contribution of arterial baroreceptors to the sense of effort.

We hypothesized that SAD would produce greater impacts on running performance and cardiovascular regulation in the warm compared with the temperate environment. In contrast to our hypothesis, there was no interaction between Ta and the SAD-mediated decrease in running time, regardless of the exercise protocol. Moreover, in both environments, the SAD-induced effects on the cardiovascular parameters occurred in the same direction; specifically, the increases in MAP, HR, and the double product were always enhanced in denervated rats. However, at 35° C, these cardiovascular differences between groups persisted until the interruption of exercise, even during the incremental-speed protocol. These long-lasting cardiovascular differences in the heat may be due to the larger blood flow supply to the cutaneous vessels, which would increase the strain on the heart and other organs involved in blood pressure regulation.

The present experiments do not allow us to precisely describe the mechanisms underlying the increased cardiovascular responses in the SAD rats. It is reasonable that a higher sympathetic outflow caused by less baroreflex inhibition may have enhanced the visceral vasoconstriction, increasing the peripheral resistance and blood pressure in both exercise protocols. Corroborating this hypothesis, a previous study reported augmented plasma norepinephrine concentrations, as well as mesenteric and renal vascular resistances, in SAD rats exposed to passive heating [20]. Further evidence for the increased sympathetic activity in our SAD rats subjected to running exercise is provided by our power spectral data, which showed an increase in the VLF and LF components of systolic pressure variability in the SAD rats compared to the SHAM rats (Table 3).

Another mechanism that may underlie the augmented pressor response of the SAD rats is a more pronounced secretion of vasopressin, as evidenced by a previous report showing that SAD rats subjected to chronic stress exhibit increased plasma concentrations of vasopressin [33]. Moreover, the results from experiments that employed pharmacological tools indicate that vasopressin contributes to increases in the MAP and to the redistribution of cardiac output during dynamic exercise [34,35].

The surgical withdrawal of the baroafferents increased the maximal HR achieved during incremental exercises (Figure 7). This higher maximal HR of the SAD rats is a consequence of their resting tachycardia but also reflects an exaggerated response to the exercise in the temperate (Δ HR: 180 ± 11 bpm vs. 134 ± 7 bpm; *P* < 0.05) and warm (Δ HR: 325 ± 12 bpm vs. 224 ± 26 bpm; *P* < 0.05) environments. Under resting conditions, the tachycardia observed in SAD rats has been associated with a decreased bradycardic vagal tonus in the heart [23]. In contrast, we hypothesize that the exaggerated exercise-induced increase in the HR could be attributed to higher cardiac sympathetic outflow. Corroborating this hypothesis, Kregel et al. [20] reported increased HR and plasma noradrenaline concentrations in SAD relative to SHAM rats when the animals were heat-exposed and presented hyperthermia, conditions that were also observed in our experiments.

The rate of increase in T_core_ during the constant-speed exercises was not modified by SAD. Interestingly, at the end of effort in the heat, the SAD rats exhibited T_core_ values 1° C lower than those of SHAM rats (Figure 4D) as a consequence of shorter exercise duration observed in the denervated rats. Because the contribution of the thermal stimuli arising from the body’s core to the fatigue sensation was diminished in denervated relative to intact rats, the increased cardiovascular strain was most likely the primary factor associated with early fatigue in the SAD animals. Additionally, these results indicate that the changes in cardiovascular responses evoked by the barodenervation are not mediated by activation of core body thermoreceptors, because SAD rats terminated the constant-speed running at 35° C with exaggerated MAP and double product (Figure 3D and 3F), despite lower T_core_ values (Figure 4D).

During the submaximal, constant-speed exercises, the denervation increased the SAP variability and the power spectral density in the VLF and LF ranges, without affecting the HR variability (Table 3). These observations indicate that the baroreflex limits blood pressure oscillations during exercise and that SAD disrupts the sympathetic-mediated cardiovascular adjustments to treadmill running. Although the mechanisms underlying the augmented SAP variability after SAD are not completely understood, this effect is strongly attenuated by intravenous injection of a sympathetic ganglionic transmission blocker [36]. Similarly, the infusion of catecholamines in conscious rats increases VLF fluctuations in the SAP spectrum through the stimulation of α_2_ receptors, suggesting that blood pressure oscillations in the VLF range have an adrenergic origin [37]. In addition, Waki [38] found a positive correlation between the lumbar sympathetic nerve activity and LF power spectrum of the SAP in resting rats, indicating that this LF component is adequate to infer the changes in vasomotor sympathetic nerve activity in rats. The lack of SAD-mediated effects on the HR variability observed in our study (Table 3) is in agreement with the previous reports indicating that HR in the rat is primarily modulated during exercise by central mechanisms responsible for coordinating the responses of the autonomic and motor systems [4].

It is important to note that our sinoaortic denervation procedure disrupted the afferent signals from the arterial baroreceptors and peripheral chemoreceptors. These chemoreceptors are the major oxygen sensors, and their stimulation increases the sympathetic vasoconstriction outflow to several vascular beds, including the skeletal muscles and visceral vessels, in rats [39,40],. Considering that a recent report implicated the carotid chemoreceptors in the exercise training-induced cardiovascular adaptations [41], we cannot exclude carotid chemoreceptors’ involvement in the cardiovascular responses evoked by an acute running exercise. Another relevant fact is that the cardiopulmonary baroreceptors were still operating in the SAD rats in this study. Because these low pressure, mechanically sensitive stretch receptors participate in the neural cardiovascular regulation during exercise [42], it cannot be stated that the denervated rats lacked all of the afferent stimuli that control blood pressure.

In conclusion, the running performance is dependent on intact arterial baroreflexes in both temperate and warm environments. In the absence of the afferent signaling from the arterial baroreceptors, the rats exhibited exaggerated increases in the HR and double product while they were running, including at the maximal power output tolerated. These findings suggest that an enhanced cardiovascular strain caused by the barodenervation at least partially accounts for the early exercise interruption in the SAD rats.

### Applicability of our findings to human physiology

The present results suggest that an impaired baroreflex and the consequent exaggerated cardiovascular strain may contribute to the lower aerobic performance in patients with diabetes, metabolic syndrome and hypertension. However, caution must be exercised before applying our findings in laboratory rats to human subjects because the two species exhibit different thermoregulatory and cardiovascular responses during passive heat exposure and during physical exercise in warm conditions. For example, humans have a greater density of eccrine sweat glands and consequently have a greater ability to dissipate heat by evaporative means while exercising [43]. With respect to the cardiovascular responses, prolonged exercise in warm and dry conditions promotes progressive decreases in the cardiac output, stroke volume and MAP in humans [44]. In contrast, laboratory rats present a progressive increase in the MAP with exercise in the heat (Figures 3D and 7D). Despite these marked thermoregulatory and cardiovascular differences, there are several physiological responses that are similar in both species, such as the exaggerated tachycardia and hyperthermia that occur during prolonged physical exercise in warm conditions [45].

In the present study, fatigue was accelerated in the SAD rats during constant and incremental-speed exercises. This early interruption of effort was associated with exacerbated increases in the MAP, HR, and VLF and LF components of SAP variability. Despite the above-mentioned interspecies differences in cardiovascular adjustments, there is evidence that enhanced cardiovascular strain also impairs human performance during prolonged exercise in the heat [46,47]. Thus, considering the large body of evidence of the role of arterial baroreceptors on the appropriate cardiovascular responses to exercise in humans [48,49], it is likely that the sensitivity of these baroreceptors may also modulate exercise performance. However, additional human studies are required to confirm this hypothesis.

Another relevant limitation for translating the present findings to human physiology is the fact that the rats were encouraged to run on the treadmill by light electrical stimulation (0.5 mA). We did not measure any objective parameters to ensure that the electrical stimulation similarly motivated the SAD and SHAM rats to maintain physical efforts. Although we cannot rule out that an interaction between the electrical stimulation and SAD may have confounded our results, previous studies observed similar changes in physical performance induced by increased central dopamine availability, regardless of whether the rats were subjected to treadmill running with [50] or without electrical stimulation [51], suggesting that this type of external prodding is not a major methodological limitation in rodent studies designed to investigate exercise fatigue. On the other hand, the cardiovascular differences between groups were observed within 5-10 min after the initiation of exercise, when the rats were running in the front of the treadmill belt, with virtually no exposure to the electrical stimulation. Moreover, our results corroborated the findings of a study that had SAD rats running at 6 m/min, without any external prodding [7]. Both investigations observed higher exercise-induced increases in the MAP, without any concomitant changes in the HR. Therefore, we can rule out the assumption that the cardiovascular differences observed between our experimental groups were due to a distinct response of SAD rats to the electrical stimulation.
